# The Substitutions L50F, E166A, and L167F in SARS-CoV-2 3CLpro Are Selected by a Protease Inhibitor *In Vitro* and Confer Resistance To Nirmatrelvir

**DOI:** 10.1128/mbio.02815-22

**Published:** 2023-01-10

**Authors:** Dirk Jochmans, Cheng Liu, Kim Donckers, Antitsa Stoycheva, Sandro Boland, Sarah K. Stevens, Chloe De Vita, Bert Vanmechelen, Piet Maes, Bettina Trüeb, Nadine Ebert, Volker Thiel, Steven De Jonghe, Laura Vangeel, Dorothée Bardiot, Andreas Jekle, Lawrence M. Blatt, Leonid Beigelman, Julian A. Symons, Pierre Raboisson, Patrick Chaltin, Arnaud Marchand, Johan Neyts, Jerome Deval, Koen Vandyck

**Affiliations:** a KU Leuven, Department of Microbiology, Immunology & Transplantation, Rega Institute, Laboratory of Virology & Chemotherapy, Leuven, Belgium; b Aligos Therapeutics, Inc., South San Francisco, California, USA; c CISTIM Leuven vzw, Leuven, Belgium; d KU Leuven, Department of Microbiology, Immunology & Transplantation, Rega Institute, Laboratory of Clinical & Epidemiological Virology, Leuven, Belgium; e Institute of Virology and Immunology, University of Bern, Bern, Switzerland; f Department of Infectious Diseases and Pathobiology, Vetsuisse Faculty, University of Bern, Bern, Switzerland; g Aligos Belgium BV, Leuven, Belgium; h Centre for Drug Design and Discovery (CD3), KU Leuven, Leuven, Belgium; i Global Virus Network (GVN), Baltimore, Maryland, USA; Icahn School of Medicine at Mount Sinai

**Keywords:** antiviral agents, coronavirus, drug resistance mechanisms, protease inhibitors

## Abstract

The SARS-CoV-2 main protease (3CLpro) has an indispensable role in the viral life cycle and is a therapeutic target for the treatment of COVID-19. The potential of 3CLpro-inhibitors to select for drug-resistant variants needs to be established. Therefore, SARS-CoV-2 was passaged *in vitro* in the presence of increasing concentrations of ALG-097161, a probe compound designed in the context of a 3CLpro drug discovery program. We identified a combination of amino acid substitutions in 3CLpro (L50F E166A L167F) that is associated with a >20× increase in 50% effective concentration (EC_50_) values for ALG-097161, nirmatrelvir (PF-07321332), PF-00835231, and ensitrelvir. While two of the single substitutions (E166A and L167F) provide low-level resistance to the inhibitors in a biochemical assay, the triple mutant results in the highest levels of resistance (6× to 72×). All substitutions are associated with a significant loss of enzymatic 3CLpro activity, suggesting a reduction in viral fitness. Structural biology analysis indicates that the different substitutions reduce the number of inhibitor/enzyme interactions while the binding of the substrate is maintained. These observations will be important for the interpretation of resistance development to 3CLpro inhibitors in the clinical setting.

## INTRODUCTION

There is an urgent need for potent and safe antiviral drugs for the treatment and prophylaxis of SARS-CoV-2 infections. Highly efficacious and safe viral protease inhibitors have contributed significantly to the effective treatment of infections with HIV and hepatitis C virus (HCV). Coronaviruses have two proteases, the main protease, 3CLpro (or Mpro), and the papain-like protease. 3CLpro is a cysteine protease that cleaves the two polyproteins (pp1a and pp1ab) of SARS-CoV-2 at 11 different sites, resulting in various nonstructural proteins, which are key for viral replication (1). The substrate of 3CLpro presents a distinct glutamine at the P1 site (Leu-Gln/Ser, Ala, Gly), while no known human proteases recognize this cleavage site (2, 3). 3CLpro can thus be considered a highly attractive drug target for the development of SARS-CoV-2 antivirals (4). The potential of 3CLpro inhibitors has become apparent with the development of nirmatrelvir (PF-07321332), a peptidomimetic reversible covalent inhibitor that is coformulated with the pharmacokinetic enhancer ritonavir (the resulting combination being marketed as Paxlovid) (5). When treatment is initiated during the first days after symptom onset, it results in substantial clinical benefit (6–9). We recently demonstrated that nirmatrelvir is equipotent *in vitro* against the current SARS-CoV-2 variants of concern (VoC). Nirmatrelvir protects Syrian golden hamsters from intranasal infection with different VoCs and prevents transmission to untreated cohoused sentinels (10, 11). Other clinical candidate 3CLpro inhibitors include ensitrelvir (S-217622), a nonpeptidic, noncovalent SARS-CoV-2 3CLpro inhibitor (12, 13), and PBI-0451 (14). The clinical development of lufotrelvir, an intravenous pro-drug of PF-00835321, has been discontinued (15, 16).

Treatment with antivirals can result in the selection of resistant viral variants and subsequent therapeutic failure. This has been described extensively in the treatment of (chronic/persistent or acute) viral infections caused by HIV, hepatitis B virus (HBV), hepatitis C virus (HCV), herpesviruses, and influenza (17, 18). Importantly, transmission of resistant viruses has been reported for HIV and influenza (19, 20). For SARS-CoV-2, selection of resistant isolates has only been described for remdesivir, a polymerase inhibitor. *In vitro* selection with remdesivir results in the emergence of resistance-associated mutations. Yet in the clinical setting, treatment with remdesivir so far only led to the selection of mutations that are associated with low-level resistance (21–23). A causal effect between SARS-CoV-2 resistance to any replicase inhibitors and therapy failure has not yet been demonstrated, most likely because these inhibitors are not yet widely used in the clinic and/or resistant variants might have a fitness disadvantage.

Here, we report on a pathway by which SARS-CoV-2 achieves significant resistance and cross-resistance to 3CLpro inhibitors during serial passage in cell culture in the presence of a first-generation 3CLpro inhibitor, ALG-097161. This molecule was prepared as a tool compound in the context of a drug discovery program.

## RESULTS

### SARS-CoV-2 acquires resistance to 3CLpro inhibitors during passage with ALG-097161.

**FIG 1:**
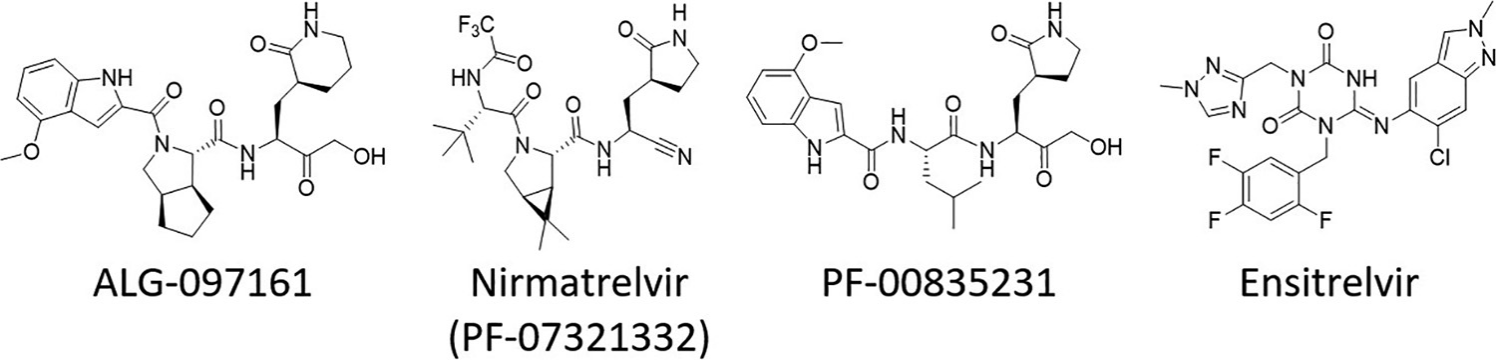
Chemical structures of different 3CLpro inhibitors.

ALG-097161 is a SARS-CoV-2 inhibitor with a 50% effective concentration (EC_50_) of 0.59 μM when tested for inhibition of SARS-CoV-2 isolate GHB-0302, a prototypic Wuhan isolate from a Belgian patient, in VeroE6 cells (Table 1). It has no effect on the viability of uninfected host cells at concentrations up to 10 μM. Since VeroE6 cells have a high efflux of some chemotypes, all selection experiments, antiviral assays, and toxicity assays on these cells were performed in the presence of the P-glycoprotein (Pgp) efflux inhibitor CP-100356 (0.5 μM) (24). The antiviral activity of ALG-097161 can be ascribed to inhibition of 3CLpro (50% inhibitory concentration [IC_50_]), 0.014 μM; Table 2), and there is no relevant inhibitory effect on human cathepsin L (IC_50_, >10 μM; see Table S1 in the supplemental material). For comparative purposes, the chemical structures of ALG-097161 and the clinical 3CLpro inhibitors nirmatrelvir, PF-00835231, and ensitrelvir are shown in Fig. 1.

**TABLE 1:** Phenotypic resistance associated with the L50F E166A L167F mutation profile as determined in VeroE6 cells^*a*^

Compound	VeroE6 CC_50_ (μM) (*n* = 4)	SARS-CoV-2 WT-GHB-03021 EC_50_ (μM) (range)	SARS-CoV-2 L50F E166A L167F EC_50_ (μM) (range) [FC]^*d*^
ALG-097161	>10	0.59^*b*^ (0.47–0.83)^*c*^ (*n* = 6)	39 [63] (13–47) (*n* = 6)
Nirmatrelvir	>10	0.12 (0.094–0.18) (*n* = 8)	6.1 [51] (5.8–8.1) (*n* = 5)
PF-00835231	>10	0.19 (0.18–0.20) (*n* = 2)	4.4 [23] (1.0–7.8) (*n* = 5)
Remdesivir (GS-441524)^*e*^	>10	0.79 (0.67–1.1) (*n* = 8)	1.7 [2.1] (0.59–1.9) (*n* = 6)

a EC_50_, 50% effective concentration; CC_50_, 50% cytostatic concentration. Note: all assays on VeroE6 cells were performed in the presence of 0.5 μM CP-100356.

b Median value.

c 25th to 75th percentile. EC_50_, 50% effective concentration.

d FC, fold change of EC_50_.

e GS-441524 is the parent nucleoside of remdesivir; it is intracellularly converted to the same active metabolite.

**TABLE 2:** Biochemical analysis of the resistance associated with different substitutions in the 3CLpro enzyme

Compound	WT IC_50_ (nM) (range)	IC_50_ (nM) [FC] (range)^*c*^		
		E166A	L167F	L50F E166A L167F
ALG-097161	14^*a*^ (8.8–17)^*b*^ (*n* = 3)	77 [5.7] (58–90) *n* = 3	72 [5.3] (55–91) (*n* = 3)	480 [35] (220–560) (*n* = 3)
Nirmatrelvir	23 (16–26) *n* = 6	230 [10] (210–380) *n* = 4	100 [4.4] (84–130) (*n* = 4)	1,600 [72] (820–1,700) (*n* = 6)
PF-00835231	13 (10–19) *n* = 3	27 [2.1] (21–47) *n* = 3	48 [3.8] (29–74) (*n* = 3)	75 [6.0] (72–210) (*n* = 3)
Ensitrelvir	25^*a*^ (17–34)^*b*^ *n* = 3	ND	ND	2300 [93] (1,422–3,002) (*n* = 4)

a Median value.

b 25th to 75th percentile.

c FC, fold change of EC_50_; ND, not determined.

10.1128/mbio.02815-22.1 TABLE S1 Enzymatic activity against 3CLpro and cathepsin L. Download Table S1, DOCX file, 0.02 MB. Copyright © 2023 Jochmans et al. 2023 Jochmans et al. https://creativecommons.org/licenses/by/4.0/ This content is distributed under the terms of the Creative Commons Attribution 4.0 International license.

**FIG 2:**
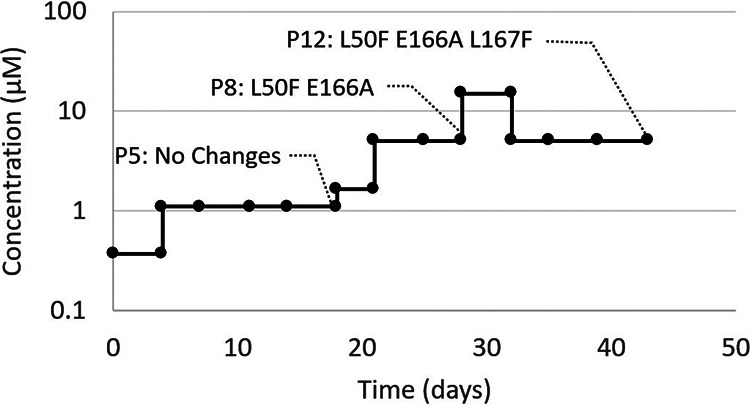
Passaging SARS-CoV-2-GHB (Wuhan) in VeroE6 cells in the presence of increasing concentrations of ALG-097161 (and the efflux inhibitor CP-100356). Selection was initiated at 0.4 μM. At every passage, several new cultures were started with the same concentration as well as a lower and a higher concentration. The passage with the highest compound concentration that could be maintained was selected for the following passage. At passages 5, 8, and 12, vRNA in the cell culture medium was sequenced. Substitutions in the 3CLpro that were found at these passages are indicated.

To identify resistance development to 3CLpro inhibitors, we passaged SARS-CoV-2-GHB-0302 in VeroE6 cells in the presence of increasing concentrations of ALG-097161. The selection started at a concentration of 0.4 μM, and for each next passage the virus was cultured at the same concentration, a 3× higher concentration, and a 3× lower concentration. The culture demonstrating virus breakthrough, as observed by a significant cytopathic effect, at the highest concentration was then used for the next passage. We started this experiment in triplicate, but for two trials we could not significantly increase the ALG-097161 concentration above 2 μM by passage 8. These cultures were therefore not further analyzed. In one culture however, the concentration of ALG-097161 could be increased gradually to 5 μM at passage 8 (p8, day 28) (Fig. 2) and maintained for one passage at 15 μM, but that concentration had to be decreased again to 5 μM for the subsequent passages to allow viral replication until p12 (day 39) (Fig. 2).

Whole-genome sequencing (Illumina) was performed on RNA purified from harvested supernatant at passages 5, 8, and 12. At p5, no mutations were identified in the 3CLpro gene, but at p8 and p12 dominant mutations (>80% of the reads) were present that result in amino acid substitutions L50F E166A and L50F E166A L167F, respectively (Fig. 2; Table 3). As a control, SARS-CoV-2 was also passaged, at the same frequency, in the absence of compound, and no mutations were identified in the 3CLpro gene at any passage (data not shown).

**TABLE 3:** Genotypic analysis of SARS-CoV-2 passaged in the presence of ALG-097161^*a*^

Nucleotide no. (NC_045512)^*c*^	Change	Gene	CDS codon no.	Gene product	3CLpro codon no.	Codon change	Amino acid change	Variant frequency (%)^*b*^		
								Passage 5	Passage 8	Passage 12
1547	A → C	ORF1ab	428	nsp2		AGC → CGC	S →s R	<10	10.4	10.0
7252	G → U	ORF1ab	2329	nsp3		UUG → UUU	L → F	17.7	18.0	18.1
7254	C → G	ORF1ab	2330	nsp3		GCA → GGA	A → G	11.5	11.6	11.8
10202	C → U	ORF1ab	3313	nsp5_3CLpro	50	CUU → UUU	L → F	<10	99.5	99.8
10551	A → C	ORF1ab	3429	nsp5_3CLpro	166	GAA → GCA	E → A	<10	88.1	99.6
10555	A → U	ORF1ab	3430	nsp5_3CLpro	167	UUA → UUU	L → F	<10	<10	98.2
15671	A → G	ORF1ab	5136	nsp12		GAG → GGG	E → G	58.1	<10	<10
16370	U → C	ORF1ab	5369	nsp13		GUU → GCU	V → A	34.5	97.7	99.0
16985	C → U	ORF1ab	5574	nsp13		ACU → AUU	T → I	<10	99.0	98.4
17057	U → A	ORF1ab	5598	nsp13		AUG → AAG	M → K	59.5	<10	<10
17332	A → G	ORF1ab	5690	nsp13		ACG → GCG	T → A	<10	80.3	98.6
21005	C → U	ORF1ab	6914	nsp16		GCA → GUA	A → V	<10	<10	99.2
21866	A → G	S	102	S		AGA → GGA	R → G	37.9	99.7	99.7
24968	A → G	S	1136	S		ACA → GCA	T → A	< 10	38.8	97.6
25538	G → U	ORF3a	49	ORF3a		GGC → GUC	G → V	< 10	78.2	97.8
26267	A → U	E	8	E		GAG → GUG	E → V	57.7	<10	<10

a The sequence information (Illumina) of the indicated passages was compared with the consensus sequence of the starting SARS-CoV-2 GHB-03021 virus. All nonsynonymous nucleotide changes that were present in >10% of the reads of any of the samples are shown.

b Color coding for variant frequency: white, <20%; light gray, 20% to 90%; dark gray, >90%.

c Note that the nucleotides encoding the 3CLpro cleavage sites are at the following positions: Nsp4-5, 10040 to 10066; Nsp5-6, 10958 to 10984; Nsp6-7, 11828 to 11854; Nsp7-8, 12077 to 12103; Nsp8-9, 12671 to 12697; Nsp9-10, 13010 to 13036; Nsp1011, 13427 to 13453; Nsp12-13, 16222 to 16248; Nsp13-14, 18025 to 18051; Nsp14-15, 19609 to 19632; Nsp15-16, 20644 to 20670.

Selection over the full genome of all nonsynonymous nucleotide changes, which were present in >10% of the reads in any of the samples, shows that other nonsynonymous mutations also became fixed (Table 3). This can be explained by the fact that the nucleotide changes, resulting in the 3CLpro substitutions, occurred in a minor variant that also had these background mutations. Importantly, all these other changes are outside the 3CLpro gene or the 3CLpro cleavage sites encoding sequences (Table 3).

For phenotypic analysis, a new virus stock (p13) was grown in the presence of 4 μM ALG-097161, and the presence of L50F E166A L167F was confirmed by genotypic analysis. Antiviral testing with this stock showed strong resistance to ALG-097161 and cross-resistance to both nirmatrelvir and PF-00835231 (Table 1). The EC_50_ values for all these 3CLpro inhibitors are increased >10×. As expected, the sensitivity of the polymerase inhibitor remdesivir remains unchanged.

In a second series of experiments, we investigated whether the observed amino acid substitutions in 3CLpro are sufficient for the resistance phenotype. To this end, we engineered, using an infectious clone (25), virus stocks with L50F, E166A L167F, or L50F E166A L167F. Unfortunately, we were not able to generate virus with E166A or L167F alone. Genotypic analysis confirmed the presence of the mutations and revealed that there were no other nonsynonymous mutations in the genome (data not shown). Antiviral testing with these engineered viruses shows that L50F by itself is not associated with resistance, while E166A L167F and L50F E166A L167F cause a 4.6× and 10.3× increase, respectively, in EC_50_ for ALG-097161 (Table 4). Again, cross-resistance is detected for nirmatrelvir (EC_50_ increased 29× with the L50F E166A L167F virus). At this stage of our study, ensitrelvir became available; we found that this molecule also shows cross-resistance (EC_50_ increased 44× with L50F E166A L167F). For all protease inhibitors tested, we observed less resistance with the E166A L167F virus than with the L50F E166A L167F virus. Importantly, all the viruses analyzed remained fully sensitive to remdesivir.

**TABLE 4:** Phenotypic resistance associated with the L50F, E166A L167F, and L50F E166A L167F mutation profiles as determined using reverse-engineered SARS-CoV-2 viruses in the USA-WA1 background on VeroE6 cells^*a*^

Compound	SARS-CoV-2 WT-USA-WA1 EC_50_ (μM) (range) (*n* = 4)	EC_50_ (μM) [FC]^*d*^ (range)		
		L50F (*n* = 4)	E166A L167V	L50F E166A L167V
ALG-97161	0.60^*b*^ (0.51–0.71)^*c*^	0.98 [1.6] (0.83–1.1)	2.9 [4.6] (1.5–4.5) (*n* = 4)	6.2 [10.3] (4.8–7.3) (*n* = 4)
Nirmatrelvir	0.11 (0.09–0.12)	0.16 [1.5] (0.12–0.2)	1.1 [10.0] (0.67–1.8) (*n* = 4)	3.2 [29] (2.3–3.5) (*n* = 6)
Ensitrelvir	0.21 (0.18–0.27)	0.24 [1.1] (0.18–0.31)	8.0 [38] (2.8–12.7) (*n* = 4)	9.3 [44] (3.5–15) (*n* = 6)
Remdesivir (GS-441524)^*e*^	0.86 (0.53–1.1)	1.1 [1.3] (0.8–1.1)	1.5 [1.7] (1.1–1.9) (*n* = 4)	1.2 [1.4] (0.91–1.7) (*n* = 6)

a EC_50_, 50% effective concentration. Note: all assays on VeroE6 cells were performed in the presence of 0.5 μM CP-100356.

b Median value.

c 25th to 75th percentile; EC_50_, 50% effective concentration.

d FC, fold change of EC_50_.

e GS-441524 is the parent nucleoside of remdesivir; it is intracellularly converted to the same active metabolite.

### Resistance to 3CLpro inhibitors in a cell-based reporter assay.

The resistance profile was next confirmed in a previously described cell-based reporter assay of SARS-CoV-2 3CLpro enzymatic function (26). In this gain-of-signal assay, inhibition of 3CLpro results in an increased enhanced green fluorescent protein (eGFP) signal. Introducing the three amino acid changes L50F E166A L167F into the construct resulted in a 23× and 28× loss of potency for ALG-097161 and nirmatrelvir, respectively (Table 5). The resistance level of the triple mutant against PF-00835231 was 6.9×.

**TABLE 5:** Phenotypic resistance associated with the L50F E166A L167F mutation profile as determined in a cell-based 3CLpro reporter assay

Compound	WT EC_50_ (μM) (range)	L50F E166A L167F EC_50_ (μM) [FC]^*c*^ (range)
ALG-097161	1.7^*a*^ (1.2–3.8)^*b*^ (*n* = 6)	39 [23] (10–39) (*n* = 3)
Nirmatrelvir	0.96 (0.65–1.2) (*n* = 5)	27 [28] (17–36) (*n* = 4)
PF-00835231	1.6 (1.2–6.0) (*n* = 6)	11 [6.9] (7.6–15) (*n* = 3)

a Median value.

b 25th to 75th percentile.

c FC, fold change of EC_50_.

### Effect of amino acid substitutions on 3CLpro enzymatic activity.

**FIG 3:**
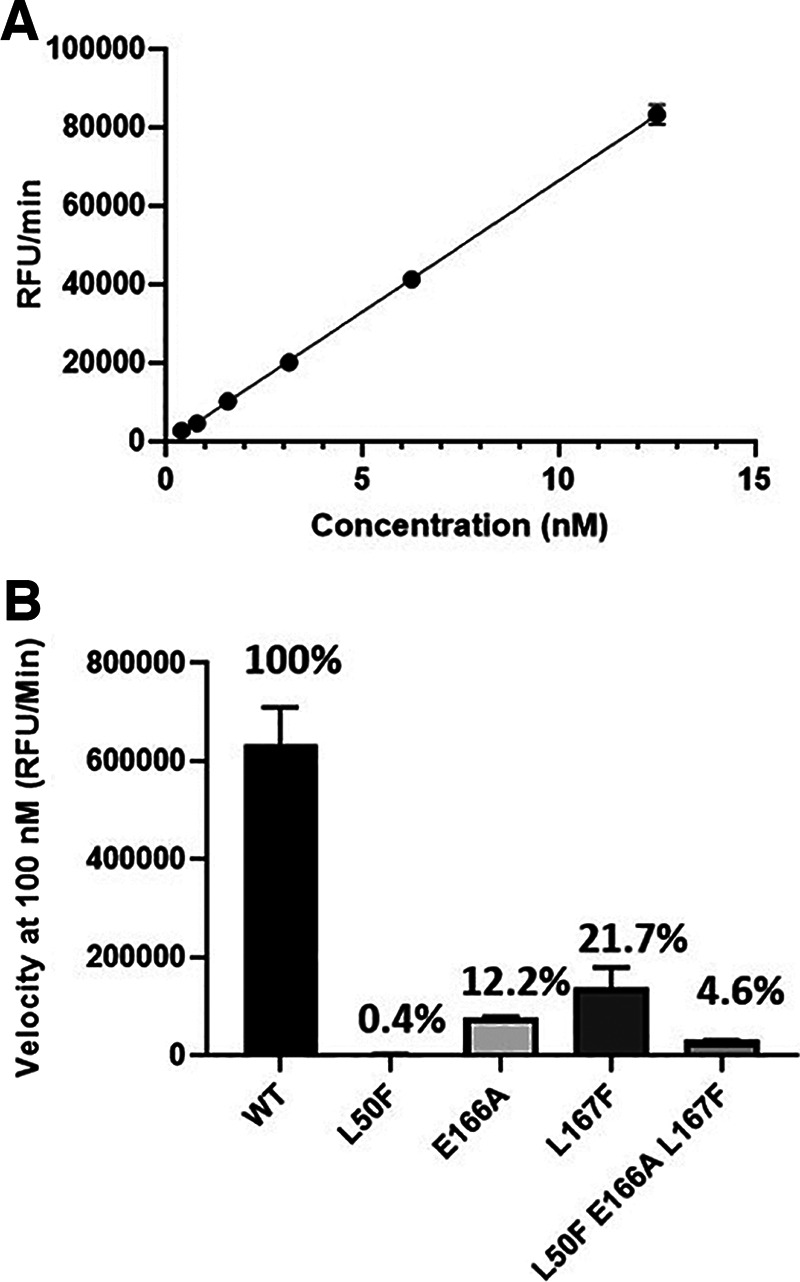
Enzymatic activity of WT and mutated SARS-CoV-2 3CLpro. (A) The enzymatic activity of WT 3CLpro was measured in a FRET assay. Three independent experiments were performed. The figure shows the results of one representative experiment. (B) Comparison of enzymatic activity between WT 3CLpro and mutated enzymes. Each data point represents the average of three independent experiments, and the mean and standard deviations are shown.

**FIG 4:**
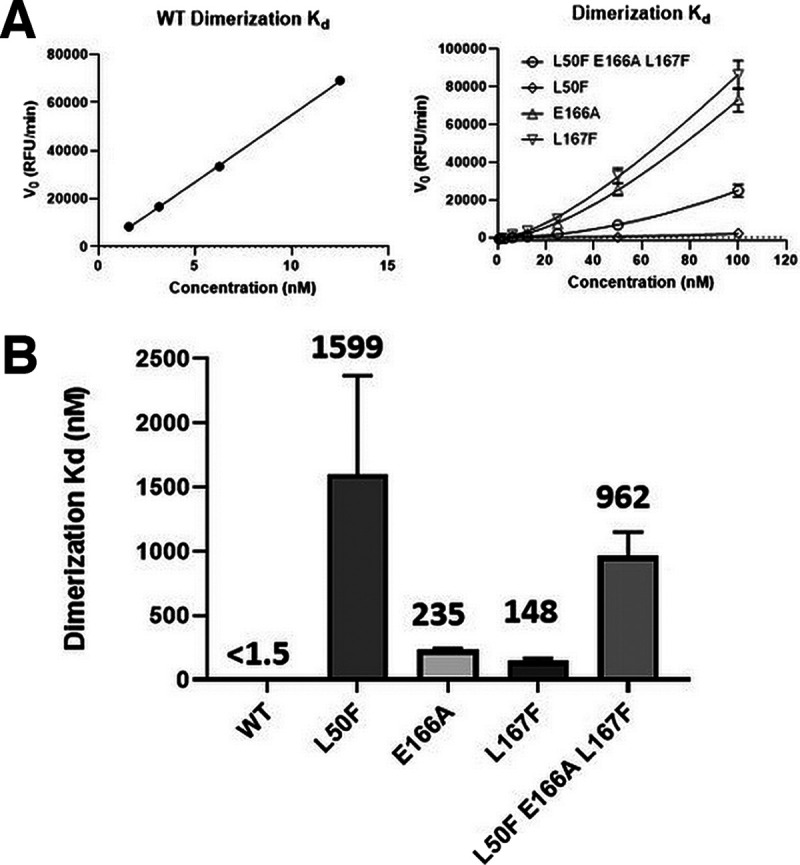
Effect of mutations on 3CLpro dimerization. (A) Initial velocities of enzyme titration of 3CLpro WT and mutant proteins were fitted as described (see Materials and Methods) to calculate the monomer dimer equilibrium dissociation constant. Three independent experiments were performed. The figure shows the results of one representative experiment. (B) 3CLpro dimerization binding affinity for each enzyme. Each data point represents the average of three independent experiments, and the mean and standard deviations are shown.

Recombinant 3CLpro proteins were produced with the L50F, E166A, and L167F substitutions alone or combined, and their enzymatic activity was tested in a fluorescence resonance energy transfer (FRET) assay using a peptide substrate featuring the canonical glutamine cleavage site (27). In this assay, wild-type (WT) 3CLpro shows linear product conversion in the enzyme concentration range of 0.5 to 12.5 nM (Fig. 3A). Compared with the WT protein, all tested mutants display reduced enzymatic activity (Fig. 3B). E166A and L167F, as single mutants, reduce the activity to about 12 to 20% of that of the wild type, whereas the L50F substitution reduces the activity to as low as 0.4%. The enzyme containing the three substitutions L50F E166A L167F is less compromised than L50F alone, with 4.6% activity compared with the WT. The loss in enzymatic activity for each mutant might be attributed to an apparent reduced binding affinity for the substrate, a loss in protein stability, and/or a reduced formation of active dimers (Fig. 4). As we tested only a single batch of recombinant protein for each mutant, we cannot exclude that some of this reduced activity is due to a lower-quality enzyme preparation.

Ligand-induced protease activation and dimerization with a low concentration of active site inhibitor has previously been described for MERS 3CLpro (28). This is explained by the inhibitor-stabilizing dimer formation by occupying only one of the two binding sites, while full occupancy of the two dimer active sites results in complete enzyme inhibition (Fig. S1). Although we did not perform direct protein dimerization studies, we observed that the impaired enzymatic activity of the mutants can be partially rescued with low concentrations (around 0.05 μM) of ALG-097161, PF-00835231, or nirmatrelvir, while higher concentrations inhibit product formation (Fig. S2).

10.1128/mbio.02815-22.2 FIG S1 Model for ligand-induced dimerization and enzyme activation at a low concentration of inhibitor. Download FIG S1, DOCX file, 0.1 MB. Copyright © 2023 Jochmans et al. 2023 Jochmans et al. https://creativecommons.org/licenses/by/4.0/ This content is distributed under the terms of the Creative Commons Attribution 4.0 International license.

10.1128/mbio.02815-22.3 FIG S2 Ligand-induced enzymatic activation with 3CLpro inhibitors. Download FIG S2, DOCX file, 0.1 MB. Copyright © 2023 Jochmans et al. 2023 Jochmans et al. https://creativecommons.org/licenses/by/4.0/ This content is distributed under the terms of the Creative Commons Attribution 4.0 International license.

### Resistance to 3CLpro inhibitors in the biochemical assay.

Based on the characterization of the different enzymes (see above), we decided to use all enzymes at a concentration of 50 nM to quantify the activity of the inhibitors. The effect of the L50F substitution could not be determined under these conditions due to its low intrinsic enzymatic activity. ALG-097161, nirmatrelvir, and PF-00835231 all inhibit WT 3CLpro, with IC_50_ values ranging from 13 to 23 nM (Table 2). For ALG-097161, the IC_50_ increases 5× to 6× with single substitutions E166A and L167F and reaches 35× for the enzyme with L50F E166A L167F. In comparison, the potency of nirmatrelvir is more affected by substitution E166A versus L167F (10× versus 4.4×) and reaches a 72× increase on the enzyme with the 3 substitutions. For PF-00835231, the shift in IC_50_ is in general lower, with a 6.0× change versus the enzyme with L50F E166A L167F. However, the shift in IC_50_ is higher (~100×) with the noncovalent and chemically distinct inhibitor ensitrelvir.

### Structural biology and computational chemistry.

Experimental data with the 3CLpro system and computational chemistry investigations were used to rationalize the phenotype observed for ALG-097161 *in vitro*.

PF-00835231 and the associated crystal structure 6XHM (24) provided a convenient starting point for modeling ALG-097161 in the catalytic pocket of 3CLpro, due to the structural similarity between the two compounds. In particular, both structures feature a hydroxymethyl-ketone warhead that binds covalently to Cys145, as well as a 4-methoxyindole-P3 substituent.

**FIG 5:**
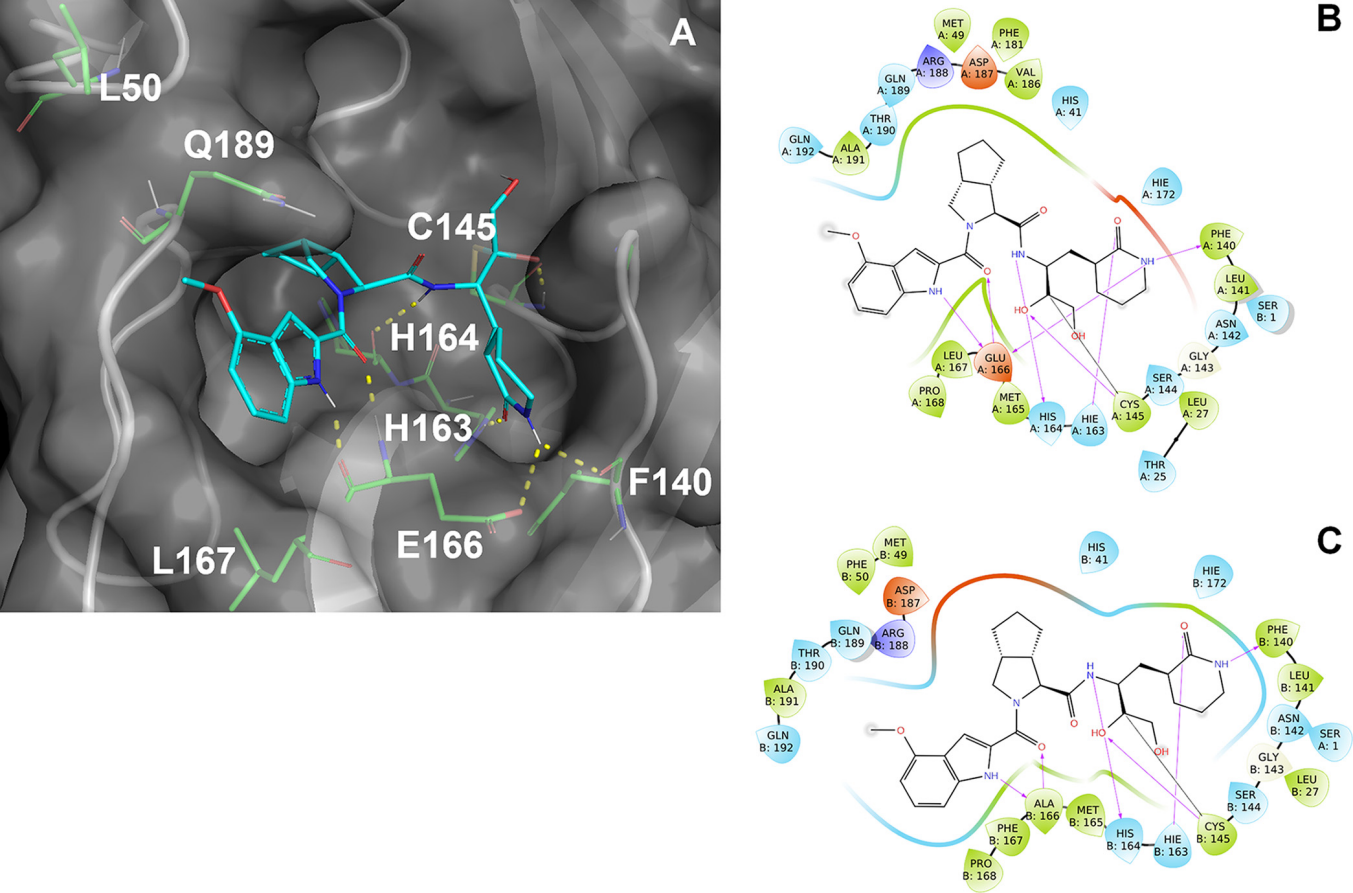
Predicted binding mode of ALG-097161 covalently bound to the catalytic site of WT 3CLpro and the effect of the L50F, E166A, and L167F substitutions. (A) ALG-097161 (carbon, cyan; nitrogen, navy; oxygen, red) covalently docked to WT 3CLpro (gray cartoon and surface; key side chains shown with carbon, green; nitrogen, navy; oxygen, red). (B) ALG-097161 binds covalently to the catalytic C145 and forms seven hydrogen bonds with 3CLpro: warhead to C145 in the oxyanion hole; P1 lactam to H163, E166, and F140; peptide backbone to H164 and E166; P3 indole to E166. The fused bicyclic P2 substitution maximizes Van der Waals interactions in the S2 subpocket. (C) Interaction diagram for ALG-097161 in triple mutant (L50F, E166A, L167F) 3CLpro. Interaction between the lactam moiety and the side chain of residue 166 is lost due to mutation, while other direct H-bond interactions are conserved (>90% occurrence) over a 100-ns MD run.

Multiple conformations of ALG-097161 were used as input for docking calculations. These yielded a well-converged predicted binding mode of ALG-097161 covalently bound to the catalytic site of WT 3CLpro (Fig. 5A). In line with the crystal structures disclosed for similar compounds (e.g., 6XHM [24]), ALG-097161 is predicted to form a covalent bond from the activated carbon atom of its hydroxymethyl-ketone warhead to the side chain of the catalytic C145 residue, anchoring the inhibitor into the binding cleft of 3CLpro. ALG-097161 forms seven direct hydrogen bonds with the SARS CoV2 3CLpro catalytic site (Fig. 5B). The oxygen atom of the inhibitor’s warhead interacts with the backbone NH of C145 in the oxyanion hole. The carbonyl oxygen of the P1 lactam hydrogen-bonds to the side chain of H163, while the NH of the P1 lactam is involved in a bifurcated interaction with the side chain of E166 and the backbone carbonyl of F140. The peptide backbone of ALG-097161 anchors itself with hydrogen bonds to the backbone carbonyl of H164 and the backbone HN of E166. The NH function of the P3 indole hydrogen-bonds the inhibitor to the backbone carbonyl of E166. The fused bicyclic P2 substituent of ALG-097161 maximizes Van der Waals interactions via extensive productive contacts to the protein side chains that form the S2 subpocket of WT 3CLpro. A key difference with PF-00835231 resides in the (lack of) interaction between the inhibitor backbone and Gln189, a direct consequence of the cyclization on P2, which results in the loss of a hydrogen bond donor site.

The predicted binding mode of ALG-097161 within 3CLpro provides some rationale for the amino acid substitutions associated with resistance (Fig. 5C). In particular, residues 166 and 167 are located within 5 Å of the bound ALG-097161. This observation can be extended to both PF-00835231 and nirmatrelvir, based on crystal structures from PDB codes 6XHM (24) and 7RFW (5), respectively. Consequently, the observed substitutions have a direct impact on the compound/target interaction.

**FIG 6:**
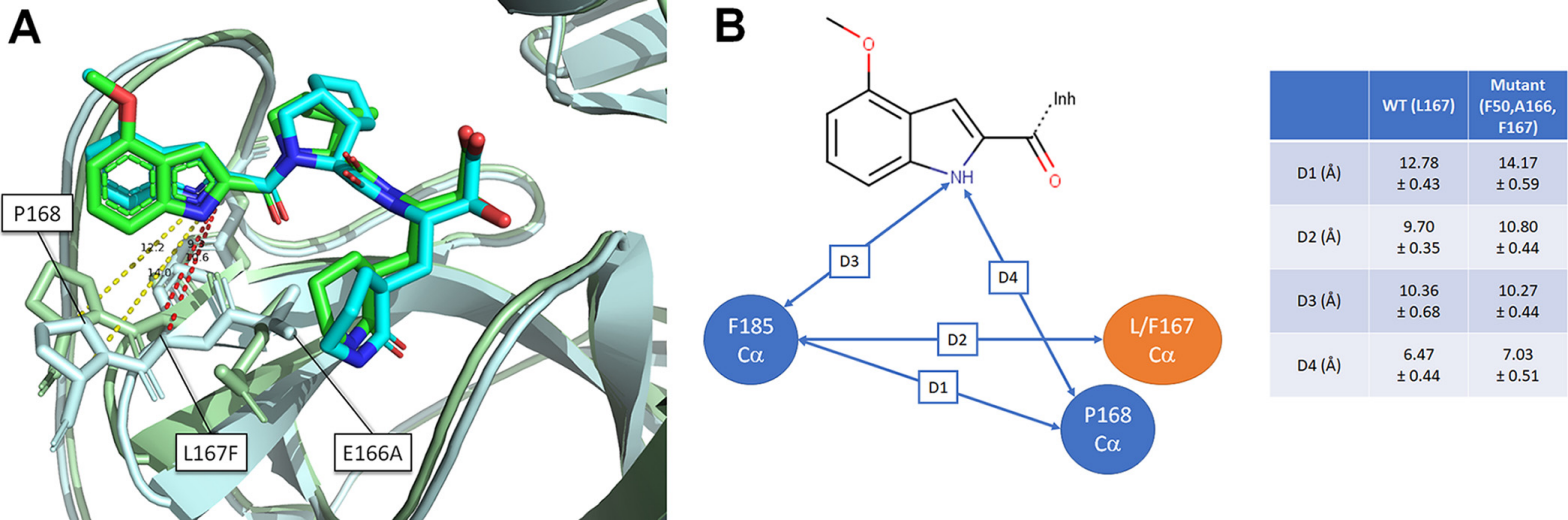
Proposed effect of the L167F substitution. Accommodation of the bulkier F167 residue results in some distortion of the binding site and in a possibly suboptimal fit. (A) Overlay of representative MD frames for WT and mutant 3CLPro. WT protein is displayed as a pale green cartoon, and the L50F E166A L167F mutant displayed as a pale blue cartoon. (B) Average distances between F185 (Cα), L167/F167 (Cα), P168 (Cα), and inhibitor. Distances were collected over 2 × 380 frames (5 ns → 100 ns simulated time). Distances between F185 and L/F167+P168 are increased following the L167F change.

E166 plays a key role in compound binding, with no less than three hydrogen bonds formed with the inhibitors. The E166A substitution eliminates one of the hydrogen bonds made within the P1 subpocket. Interactions with this subpocket are important for substrate recognition by coronaviral 3CLpro enzymes, and its occupancy, in many cases by a lactam moiety, is a key driver for inhibitor potency. It is therefore unsurprising that all three inhibitors are adversely affected by the E166A change (Fig. 5C). Meanwhile, L167F results in the presence of a bulkier side chain on the neighboring position. While this change does not directly conflict with compound binding, the side chain of L167 is in close contact with other residues, including Phe185 (Fig. 6). Molecular dynamics (MD) simulations on the L50F E166A L167F enzyme suggest that the bulkier Phe side chain results in some distortion of the neighboring subpocket. In particular, the loop containing L167F moves away from F185, and the distance between the Cαs of F185 and L167F/P168 consistently increases, resulting in a more open binding site and a suboptimal fit of the inhibitor, even though H-bond interactions (including the two remaining H-bonds with the A166 backbone) appear conserved throughout 100-ns simulations (Fig. 6). Interestingly, all three inhibitors (ALG-097161, PF-00835231, and nirmatrelvir) were affected by the L167F change.

The reasons behind the L50F mutation remain more elusive, as this residue falls outside direct Van der Waals or hydrogen-bond contact range to ALG-097161. However, the L50 side chain is located only 2.7 Å from Q189, a residue which can interact with the peptide backbone of this inhibitor class either directly (as with PF-00835231) or via water molecules (Fig. 5). L50F might therefore indirectly affect compound binding by altering the position of Q189 and the subsequent interactions mediated by this residue. However, a rigorous assessment of how this change impacts enzyme activity and/or inhibitor binding would require more elaborate techniques (e.g., free energy perturbation) that are beyond the scope of this work.

Finally, it is anticipated that the natural SARS CoV-2 polyprotein substrates, which are processed by the 3CLpro, are less affected by the L50F, E166A, and/or L167F substitutions, due to their larger size and approximately 2-fold higher number of polar interactions with the protease (as observed in 2Q6G for SARS CoV-1 3CLpro in complex with bound substrate).

## DISCUSSION

Nirmatrelvir is the first oral SARS-CoV-2 (3CLpro) protease inhibitor granted emergency use authorization by the U.S. FDA for the treatment of COVID-19. To avoid resistance development, HIV and HCV protease inhibitors are used, with great success, in fixed-dose combinations with other directly acting antiviral drugs that have a different mechanism of action (29). As a single agent, several of these inhibitors select (rapidly) for drug-resistant variants. SARS-CoV-2 infections are typically acute in nature; hence, treatment can be of short duration, which may largely reduce the likelihood that resistant variants develop. Yet, also for acute infections, the emergence of drug-resistant variants is a serious concern, certainly in case that such variants can efficiently transmit, resulting in a loss of therapeutic options for patients. For example, the first-generation influenza drugs, amantadanes, are no longer recommended, as resistance to amantadine and rimantadine is widespread (20).

To monitor clinical drug resistance, it is crucial to identify the mutations involved. To that end, we used the 3CLpro inhibitor ALG-097161, a probe compound in the context of a drug discovery program, to select for resistant variants *in vitro* by passaging SARS-CoV-2 in the presence of increasing drug concentrations. Similar experiments have been performed with SARS-CoV-2 neutralizing antibodies and polymerase inhibitors (22, 30).

The selection process indicated that there is a significant barrier to resistance for the 3CLpro inhibitor ALG-097161. The first changes were observed only between passages 5 and 8 (20 to 30 days). These were the double substitution L50F E166A in 3CLpro, which then evolved to the triple substitution L50F E166A L167F. Phenotyping this triple mutant virus revealed a 63× increase in the EC_50_ for ALG-097161. Moreover, significant cross-resistance (>10× increase EC_50_) was observed with nirmatrelvir, the active component of Paxlovid, and PF-00835231, an earlier 3CLpro clinical candidate. Next, we engineered an infectious clone with the only the 3Clpro substitutions, L50F E166A L167F, and the resistance against ALG-097161, nirmatrelvir and also ensitrelvir could be confirmed (>10× increase in EC_50_). As a second confirmation, a cell-based assay was used with heterologous expression of wild-type and mutated 3CLpro (L50F E166A L167F) and a substrate linked to a reporter protein. The EC_50_ values for ALG-097161 and nirmatrelvir in this assay increased 23× and 28×, respectively, compared with the wild type. Importantly, all of the viruses analyzed remained fully susceptible to the polymerase inhibitor remdesivir (GS-441524 form).

We further characterized the 3CLpro substitutions using biochemical assays. Enzymes carrying the single substitution L50F, E166A, or L167F were found to have markedly lower enzymatic activity (<20% compared to the wild type). Each of the enzymes was also less efficiently inhibited by the 3CLpro inhibitors. The largest increase in IC_50_ was observed when all three substitutions, L50F E166A L167F, were combined. For this triple mutant enzyme, the IC_50_ for ALG-097161, nirmatrelvir, and ensitrelvir increased by 35×, 72×, and 93×, respectively.

A structural biology analysis reveals that the substitutions decrease interactions between the enzyme and the inhibitor. E166A causes a direct loss of a hydrogen bond with the lactam moiety that is found in all the inhibitors tested here, L167F increases the size of the binding pocket, causing a decrease of the Van der Waals forces between enzyme and inhibitor, and L50F is thought to change the rotamer conformation of Q189, which, for the wild type, has many interactions with the inhibitors through a network of water molecules.

The loss of interactions, resulting from amino acid substitutions associated with resistance, might have a lower effect on substrates than on inhibitors, as the former rely on a significantly higher number of interactions for binding. Consequently, these resistance-associated mutations seem to allow a better discrimination between substrate and inhibitor at the expense of intrinsic enzymatic activity.

A recent sequence analysis across 4.9 million global SARS-CoV-2 isolates, circulating prior to the introduction of Paxlovid, showed a high genetic conservation of the 3CLpro protein (31). From the 306 amino acid positions, only 3 have a polymorphism in >1% of the samples, and 13 have a change in 1% to 0.11% of the samples. Changes at position L50, E166, or L167 have a <0.11% prevalence. E166 displays extreme high conservation in this data set, with <0.001% samples having an alternative amino acid.

At this moment, peer-reviewed information on resistance against 3CLpro inhibitors in COVID-19 patients is, to our knowledge, not available. The Paxlovid label indicates that E166V is more common in nirmatrelvir/ritonavir-treated subjects relative to placebo-treated subjects (0.8% and <0.2%, respectively), and in one patient, with a baseline L50F substitution, the E166V substitution cooccurred with L50F on day 5 of treatment (32). Both these observations are in accordance with the results described above.

While we can clearly observe resistance of different 3CLpro inhibitors for the L50F E166A L167V virus, we could not fully characterize the contribution of each substitution by itself. During the selection experiment, L50F together with E166A was selected first, and L167V was acquired later. Additional cell-based experiments demonstrate that an L50F-carrying virus is not resistant, while biochemical experiments indicate that E166A and L167F are associated with resistance. Taking these findings together, we hypothesize that E166A is the main driver for resistance, that L50F is needed to support E166A, and that the presence of L167F further increases resistance. It would require an extensive study of the replication capacity of the different variants to support this hypothesis. Importantly, clear nirmatrelvir/ensitrelvir cross-resistance is observed with both the E166A L167F and L50F E166A L167F viruses.

During the writing of this manuscript, two other studies on the selection of nirmatrelvir-resistant SARS-CoV-2 viruses were reported. One group performed >480 independent selection experiments, resulting in the identification of multiple pathways of nirmatrelvir resistance (33). They conclude that E166V confers the highest level of resistance of all substitutions tested (100× for nirmatrelvir; 23× for ensitrelvir). In addition, these authors show that E166V causes a reduced fitness that is restored by adding L50F or T21I. The selection of L50F E166V by nirmatrelvir *in vitro* has also been reported by another group (34). Also, these authors come to the conclusion that E166V is responsible for resistance, whereas L50F is a compensatory substitution required for maintaining fitness. As E166A and E166V are very similar substitutions (alanine and valine have similar chemical structures), these findings are in strong agreement with our observations. A third group engineered a chimeric vesicular stomatitis virus (VSV)-variant, which has SARS-CoV-2 3CLpro expressed in an artificial polyprotein, for which the replication depends on 3CLpro activity. Selection for nirmatrelvir resistance resulted in the appearance of L167F in the absence of other mutations, and an engineered SARS-CoV-2 infectious clone with only the L167F substitution showed a marginal level of resistance for nirmatrelvir (2× increase EC_50_) (35).

Important to mention is also the work of Flynn et al. (36), who performed different functional 3CLpro screens in yeast to study the resilience of all amino acid positions toward substitutions. Interestingly, the authors conclude that position E166 is highly tolerant to substitutions including the E166A or E166V substitution. Based on available structural biology data of 3CLpro in complex with inhibitors or natural substrate and their functional screen data, these authors identified E166A and E166Q as potentially resistance-associated substitutions.

Our report emphasizes the need for additional research to elucidate potential resistance pathways of SARS-CoV-2 inhibitors *in vitro*. It is also a starting point for the surveillance of nirmatrelvir resistance and indicates the potential need for combination therapies with different classes of SARS-CoV-2 inhibitors.

## MATERIALS AND METHODS

### Compounds, cells, viruses, proteins, and peptides.

ALG-097161, nirmatrelvir, and PF-00835231 were synthesized by Aligos Therapeutics and purified to >95% purity. The synthesis of ALG-097161 is described in Text S1. GS-441524 was obtained from MedChem Express (catalog [cat.] no. HY-103586). The African green monkey kidney VeroE6 cell line was purchased from ATCC (cat. no. CRL-1586) and maintained in Dulbecco’s modified Eagle’s medium (DMEM; Gibco cat. no. 41965-039) supplemented with 10% (vol/vol) heat-inactivated fetal calf serum (FCS).

10.1128/mbio.02815-22.4 TEXT S1 Chemical synthesis of ALG-097161. Download Text S1, DOCX file, 0.1 MB. Copyright © 2023 Jochmans et al. 2023 Jochmans et al. https://creativecommons.org/licenses/by/4.0/ This content is distributed under the terms of the Creative Commons Attribution 4.0 International license.

The SARS-CoV-2 GHB-03021 (EPI ISL407976|2020-02-03) isolate was obtained from a Belgian patient returning from Wuhan in February 2020. The isolate was passaged 7 times on VeroE6 cells, which introduced two series of amino acid deletions in the spike protein (37).

SARS-CoV-2 3CLpro wild-type and mutant enzymes were produced as previously described (38). Peptide substrate (Dabcyl-KTSAVLQSGFRKM-E(Edans)-NH_2_) for FRET was sourced from Biopeptide (San Diego, CA) at >95% purity.

### Genotypic analysis.

**(i) Illumina sequencing.** RNA was extracted from cell culture supernatant using the NucleoSpin RNA virus kit (Macherey-Nagel) according to the manufacturer’s instructions. Whole-genome sequencing (WGS) was outsourced to Eurofins Genomics (ARTIC SARS-CoV-2 WGS, Konstanz, Germany), who performed reverse transcription, enrichment of the viral genome using a primer set similar to the ARTIC primers (>200 primer pairs, covering the full 29.9-kb viral genome), generation of libraries, Illumina sequencing (2 × 150-bp read mode), and sequence cleaning to remove adapters and poor-quality bases. Sequences were further analyzed using Geneious Prime software (v2022.2.1) by mapping to the SARS-CoV-2 reference sequence (RefSeq; NC_045512), and variant calling was performed as described by the software manufacturer (https://help.geneious.com/hc/en-us/articles/360045070991-Assembly-of-SARS-CoV-2-genomes-from-tiled-amplicon-Illumina-sequencing-using-Geneious-Prime). For each sample, >0.9 million (M) reads that could be aligned with the SARS-CoV-2 genome were obtained, with >99.5% of untrimmed bases being of high quality and with a mean coverage of >4,000 reads.

**(ii) Oxford Nanopore sequencing.** As a control, the same samples were also sequenced using an inhouse pipeline. For this, reverse transcription was carried out via SuperScript IV, and cDNA was posteriorly amplified using Q5 high-fidelity DNA polymerase (NEB) with the ARTIC nCov-2019 primers, following the recommendations in the sequencing protocol of the ARTIC Network (https://artic.network/ncov-2019). Samples were multiplexed following the manufacturer’s recommendations using the Oxford Nanopore native barcoding expansion kits NBD104 (1-12) and NBD114 (13-24), in conjunction with ligation sequencing kit 109 (Oxford Nanopore). Sequencing was carried out on a MinION sequencer using R9.4.1 flow cells and MinKNOW 2.0 software. Both methods resulted in the same consensus sequence for each sample. For further mutation analysis, we used the deep sequencing data generated by the Illumina protocol.

### Antiviral testing.

The 50% effective concentration (EC_50_), the concentration of compound required for 50% antiviral activity, was determined on VeroE6 cells as follows. On day -1, the test compounds were serially diluted in 100 μL assay medium (DMEM supplemented with 2% [vol/vol] FCS) in 96-well plates. In the next step, 50 μL of VeroE6 cells (25,000 cells/well) was added together with 2 μM the MDR1-inhibitor CP-100356 (final concentration 0.5 μM). The plates were incubated (37°C, 5% CO_2_, and 95% relative humidity) overnight. On day 0, 50 μL of SARS-CoV-2 WT or the triple mutant strain at a multiplicity of infection (MOI) of 0.001 tissue culture infectious dose (TCID_50_) per cell was added, and the plates were stored in a humidified incubator at 37°C and 5% CO_2_. In the absence of antiviral activity, the VeroE6 cells underwent a cytopathic effect. Cell viability was determined 4 days postinfection (p.i.) using viability staining with MTS (3-(4,5-dimethylthiazol-2-yl)-5-(3-carboxymethoxyphenyl)-2-(4-sulfophenyl)-2H-tetrazolium) (39). The percentage of antiviral activity was calculated by subtracting the background and normalizing to the untreated-uninfected control wells, and the EC_50_ was determined using logarithmic interpolation.

### Generation and rescue of recombinant viruses.

We used the in-yeast transformation-associated recombination (TAR) cloning method as described previously with some adaptations (25). In sum, the whole SARS-CoV-2 genome was encoded in 12 overlapping DNA fragments. These so-called WU-fragments and a TAR-vector were homologously recombined in yeast, forming the yeast artificial chromosome (YAC). The SARS-CoV-2 3CLpro was encoded on WU-fragment 5 and was replaced with newly generated and overlapping PCR products to introduce the amino acid changes L50F, E166A L167F, or L50F E166A L167F. The overlapping PCR products were made via reverse transcription from RNA purified from viral RNA (vRNA) of the virus stock obtained from the selection. In brief, cDNA was generated from RNA by LunaScript RT SuperMix (NEB). PCRs targeting the 3CLpro gene were performed using Q5 high-fidelity DNA polymerase (NEB), and the resulting PCR products were mixed and matched to recombine SARSCoV-2 3CLpro^L50F^, SARSCoV-2 3Clpro^E166A L167F^, and SARSCoV-2 3CLpro^L50F E166A L167F^. All PCR products were purified with the high pure PCR product purification kit (Roche) before being used for TAR cloning.

*In vitro* transcription was performed for EagI-cleaved YACs and the PCR-amplified SARS-CoV-2 N gene using the T7 RiboMAX large-scale RNA production system (Promega) as described previously (25). Transcribed capped mRNA was electroporated into baby hamster kidney (BHK-21) cells expressing SARS-CoV N protein. Electroporated cells were cocultured with susceptible Vero TMPRSS2 cells to produce passage 0 (P.0) of the recombinant viruses. Passage 1 virus stocks were further produced in Vero human transmembrane serine protease 2 (TMPRSS2) cells, and the presence of mutations was confirmed by Illumina sequencing. These results also showed the absence of other nonsynonymous mutations in the viral genome.

### 3CLpro cell-based reporter assay.

The cell-based reporter assay of SARS-CoV-2 3CLpro enzymatic function was described previously (26). This gain-of-signal assay is based on the expression of a chimeric protein consisting of Src-Mpro-Tat-eGFP in which the Mpro amino acid sequence is flanked with cognate N- and C-terminal self-cleavage sites. While it was expected that Mpro inhibition would result in a different subcellular localization of the GFP signal, it was found that without Mpro inhibition, there is no detectable GFP expression, while in the presence of Mpro inhibition, there is high GFP expression—hence, the name gain-of-signal assay. The exact mechanism behind this reporter system is not known, but results suggest a mechanism in which Mpro activity somehow suppresses the accumulation of reporter mRNA in transfected cells.

293T cells were maintained at 37°C/5% CO_2_ in DMEM (Corning, cat. no. 10-013-CV) supplemented with 10% fetal bovine serum (Gibco, cat. no. 10091148) and 1% penicillin/streptomycin (Gibco, cat. no. 15140122). 293T cells were seeded in a 96-well plate at 5 × 10^5^ cells/well and returned to incubation for 24 h. The following day, cells were treated with compound at a final dimethyl sulfoxide (DMSO) concentration of 2% prior to transfection with 100 ng of the wild-type or triple mutant (L50F E166A L167F) plasmid with Lipofectamine LTX (Thermo Fisher, cat. no. 15338100) for 24 h. Then, 24 h posttransfection, GFP fluorescence was detected with a Victor device (Perkin Elmer) using the fluorescein 485/535 setting. Inhibition was determined from the resulting fluorescent signal and graphed using a variable slope four-parameter curve fitting with GraphPad Prism (v9.2.0).

### 3CLpro FRET-based assay.

The FRET-based assay was performed similarly to the self-assembled monolayers for matrix-assisted desorption/ionization mass spectrometry (SAMDI-MS) assay. Assays were performed in 20-μL volume in 384-well nonbinding low-volume plates (Greiner Bio-One, Monroe, NC) at ambient temperature. 3CLpro and its mutants were preincubated with inhibitors for 30 min. Reactions were initiated by the addition of a FRET-compatible peptide substrate, dabcyl-KTSAVLQSGFRKM-E(Edans)-NH_2_. Fluorescence was measured for 90 min at 2-min intervals using 340/460-excitation/emission filters on an Envision plate reader (Perkin Elmer). The IC_50_ values were calculated by fitting the curves using a four-parameter equation in GraphPad Prism.

To calculate the dimer dissociation constant (*K_d_*), the velocities of enzyme titration of WT and mutant 3CLpros were fitted to equations 1 and 2.

(1) $$V_{0}=V_{\max}[S]/(\mathrm{Km}\;+\;[S])$$

(2) $$V_{\max}=K_{\mathrm{cat}}\left[D\right]=K_{\mathrm{cat}}\frac{K_{d}\;+\;4C_{T}\;-\;\sqrt{K_{d}{}^{2}\;+\;8K_{d}C_{T}}}{8}$$

Equation 2 was described previously for the calculation of the monomer dimer equilibrium dissociation constant (*K_d_*) (28).

### Modeling of inhibitors in WT and mutant (L50F E166A L167F) 3CLpro.

PDB structure 6XHM (complex with PF-00835231) was used as the starting point for modeling compounds in WT and mutant (L50F E166A L167F) 3CLpro. The receptor structure was cleaned up and minimized with restraints ahead of docking using the Protein Preparation Wizard in Maestro (Schrödinger). Ligands were processed using the Ligprep module in Maestro. Stereoisomers were not generated, as the absolute configuration of the ligand was known.

Covalent docking was used to predict the binding mode of ALG-097161. C145 was specified as the reactive residue for covalent binding, and the centroid of the X-ray reference ligand was used to define the investigated binding site. A custom covalent reaction parameter file was used to define the reaction between the warhead moiety and the catalytic C145. A high-accuracy (pose prediction thorough) docking methodology was implemented for pose prediction. Initial docking poses within 4 kcal/mol (glide gscore) were collected for further processing. Residues within 6 Å of the bound compound were minimized after covalent bond formation. The top 5 binding mode suggestions were saved for visual inspection.

Initial structures for the L50F E166A L167F triple mutant were built by introducing the mutations directly into the WT complex. An initial restrained minimization was done with the protein preparation wizard to remove any unacceptable clash. Next, a Desmond simulation system was built (see below) and a 100-ps energy minimization procedure was run with Desmond to reach the nearest energy minimum.

Molecular dynamics (MD) simulations were performed using the Desmond-GPU package. A simulation system was first set up around the protein-inhibitor complex, including a 10-Å water box, Na^+^ ions necessary to neutralize the system, and additional Na^+^ and Cl^–^ ions to simulate a 0.15 M NaCl concentration. OPLS4 was selected as the force field. The NPT ensemble (temperature [*T*] = 300 K, *P* = 1.01325 bar) was used. The system was relaxed prior to simulation. MD simulations were run for a (simulated) period of 100 ns, with frames saved every 0.25 ns (total, 400 frames). The final frames were energy-minimized (100-ps minimization protocol). Protein-ligand interactions and distances were evaluated in the 5-ns to 100-ns period.
