# Supplementary material for: The Substitutions L50F, E166A, and L167F in SARS-CoV-2 3CLpro Are Selected by a Protease Inhibitor In Vitro and Confer Resistance To Nirmatrelvir
Source: mBio. 2023 Jan 10;14(1):e02815-22. doi: 10.1128/mbio.02815-22 (PMC9973015; doi:10.1128/mbio.02815-22)
Supplement: TABLE S1 [file mbio.02815-22-s0001.docx]

**Supplemental Table S1: Enzymatic data against 3CLpro and Cathepsin L**

|  | **IC_50_ (nM)** | |
| --- | --- | --- |
|  | **3CLpro** | **Cathepsin L***** |
| **ALG-097161** | 14*  (8.8-17)** n=3 | > 10,000  n=1 |
| **Nirmatrelvir** | 23  (16-26) n=6 | > 10,000  n=1 |
| **PF-00835231** | 13  (10-19) n=3 | 130  (61 – 260) n=58 |
| **Ensitrelvir** | 25*  (17-34)** n=3 | > 10,000  n=1 |

* Median value

** 25th – 75th percentile

***Cathepsin L assay was performed as described previously (Liu, C., et al., Antiviral Res, 2021. 187: p. 105020.). In this endpoint assay the signal was measured at 30 min after initiation.
