# Supplementary material for: The Substitutions L50F, E166A, and L167F in SARS-CoV-2 3CLpro Are Selected by a Protease Inhibitor In Vitro and Confer Resistance To Nirmatrelvir
Source: mBio. 2023 Jan 10;14(1):e02815-22. doi: 10.1128/mbio.02815-22 (PMC9973015; doi:10.1128/mbio.02815-22)
Supplement: FIG S1 [file mbio.02815-22-s0002.docx]

**Supplemental Fig S1:**

**
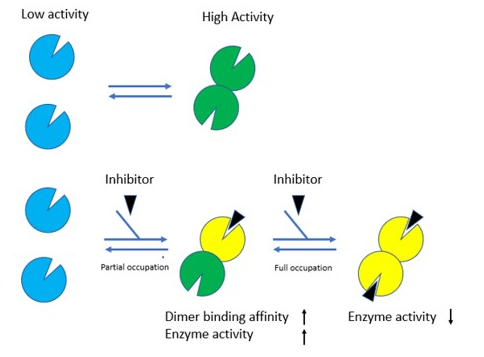
**

**Model for ligand-induced dimerization and enzymatic activation at low concentration of inhibitor.**
