# Supplementary material for: The Substitutions L50F, E166A, and L167F in SARS-CoV-2 3CLpro Are Selected by a Protease Inhibitor In Vitro and Confer Resistance To Nirmatrelvir
Source: mBio. 2023 Jan 10;14(1):e02815-22. doi: 10.1128/mbio.02815-22 (PMC9973015; doi:10.1128/mbio.02815-22)
Supplement: FIG S2 [file mbio.02815-22-s0003.docx]

**Supplemental Figure S2**

A


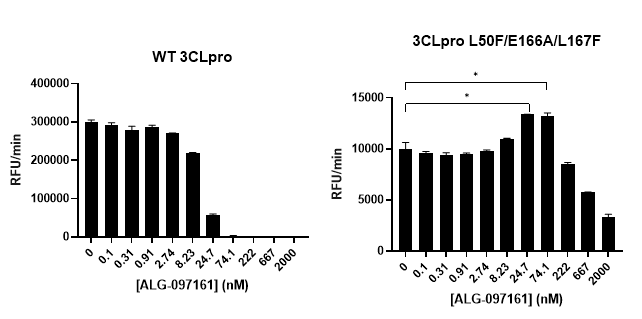


B


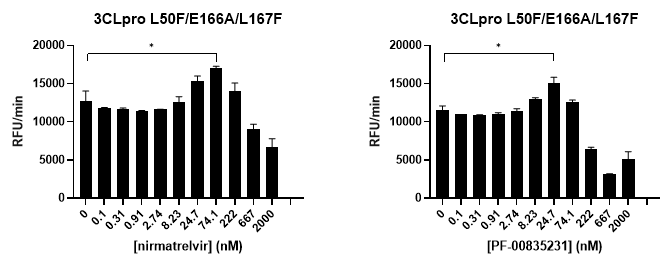


**Ligand-induced enzymatic activation.** (A) Comparison of ALG-097161 dose-response for WT and L50F E166A L167F 3CLpro. Three independent experiments were performed and mean and standard deviations are shown. The figure shows the results of one representative experiment. (* P< 0.05). (B) Nirmatrelvir and PF-00835231 dose-response for L50F E166A L167F 3CLpro. (* P< 0.05)
