# Supplementary material for: The Substitutions L50F, E166A, and L167F in SARS-CoV-2 3CLpro Are Selected by a Protease Inhibitor In Vitro and Confer Resistance To Nirmatrelvir
Source: mBio. 2023 Jan 10;14(1):e02815-22. doi: 10.1128/mbio.02815-22 (PMC9973015; doi:10.1128/mbio.02815-22)
Supplement: TEXT S1 [file mbio.02815-22-s0004.docx]

**Supplemental Text S1: Chemical synthesis of ALG-097161**

**Scheme S1. Synthesis of Intermediate 4*^a^***

*^a^*Reagents and conditions: (i) HATU, DIPEA, DMF, 78%. (ii) TFA, CH_2_Cl_2_, quantitative.

***tert*-Butyl (1*S*,3a*R*,6a*S*)-2-(4-methoxy-1*H*-indole-2-carbonyl)octahydrocyclopenta[*c*]pyrrole-1-carboxylate (3)**

A mixture of 4-methoxy-1*H*-indole-2-carboxylic acid (348 mg, 1.82 mmol), HATU (693 mg, 1.82 mmol) and DIPEA (0.866 mL, 4.97 mmol) in DMF (5 mL) was stirred at rt for 30 min. *tert*-Butyl (1*S*,3a*R*,6a*S*)-octahydrocyclopenta[*c*]pyrrole-1-carboxylate oxalate (500 mg, 1.65 mmol) was added and the reaction mixture was stirred at rt for 3 h. The reaction mixture was diluted with water (20 mL) and was extracted with EtOAc (3 x 50 mL). The organic phases were combined, washed with brine (2 x 30 mL), dried over Na_2_SO_4_, filtered and concentrated under reduced pressure. The residue was purified by flash chromatography on silica gel using a gradient of EtOAc (0-30%) in petroleum ether to afford 530 mg (78%) of the title compound as a white solid. LCMS (ESI, m/z): 329 [M-56+ H]^+^.

^1^H NMR (300 MHz, DMSO-*d*_6_) δ 11.54 - 11.65 (m, 1H), 7.08 - 7.18 (m, 1H), 7.00 - 7.07 (m, 1H), 6.88 - 6.99 (m, 1H), 6.46 - 6.56 (m, 1H), 4.21 - 4.34 (m, 1H), 4.01 - 4.18 (m, 1H), 3.84 - 3.96 (m, 3H), 3.69 - 3.82 (m, 1H), 2.52 - 2.94 (m, 2H), 1.49 - 2.05 (m, 6H), 1.28 - 1.47 (m, 9H).

**(1*S*,3a*R*,6a*S*)-2-(4-methoxy-1*H*-indole-2-carbonyl)octahydrocyclopenta[*c*]pyrrole-1-carboxylic acid (4)**

To a solution of **3** (200 mg, 0.520 mmol) in CH_2_Cl_2_ (3 mL) cooled at 0°C was added TFA (1 mL). The reaction mixture was stirred at rt for 1 h and was concentrated under reduced pressure to afford quantitatively the title compound which was used in the next step without further purification. LCMS (ESI, m/z): 329 [M+H]^+^.

**Scheme S2. Synthesis of Intermediate 9*^a^***

*^a^*Reagents and conditions: (i) 3M NaOH, MeOH, 60%. (ii) *N*,*O*-Dimethylhydroxylamine hydrochloride, NMM, HOBt, ECDI, CH_2_Cl_2_, 66%. (iii) Mg, HgCl_2_, benzylchloromethyl ether, THF, 28%. (iv) 4N HCl in dioxane, dioxane, quantitative.

**(*S*)-2-((*tert*-Butoxycarbonyl)amino)-3-((*S*)-2-oxopiperidin-3-yl)propanoic acid (6)**

To a solution of methyl (*S*)-2-((*ter*t-butoxycarbonyl)amino)-3-((*S*)-2-oxopiperidin-3-yl)propanoate (3.00 g, 10.0 mmol, synthesized according to *J. Med. Chem*., **2015**, 9414-9420) in MeOH (15 mL) cooled at 0°C was added 3M NaOH (15 mL, 45.0 mmol). The reaction mixture was stirred at 0°C for 1 h. The reaction mixture was partially concentrated under reduced pressure to remove MeOH. The residue was extracted with EtOAc (3 x 30 mL). The organic phases were combined, washed with brine (2 x 20 mL), dried over Na_2_SO_4_, filtered and concentrated under reduced pressure to provide 1.70 g (60%) of the title compound as a white solid. . ^1^H NMR (300 MHz, DMSO-*d*_6_) δ 12.49 (br s, 1H), 7.47 (s, 1H), 7.22 (d, *J* = 8.3 Hz, 1H), 3.88 - 4.05 (m, 1H), 3.09 - 3.11 (m, 2H), 2.07 - 2.16 (m, 2H), 1.82 - 1.96 (m, 1H), 1.68 - 1.83 (m, 1H), 1.57 - 1.62 (m, 2H), 1.38 - 1.40 (m, 10H). LC-MS (ESI, m/z): 287 [M+H]^+^.

***tert*-Butyl ((*S*)-1-(methoxy(methyl)amino)-1-oxo-3-((*S*)-2-oxopiperidin-3-yl)propan-2-yl)carbamate (7)**

To a mixture of **6** (1.70 g, 5.93 mmol) in CH_2_Cl_2_ (25 mL) cooled at 0°C were added *N*,*O*-dimethylhydroxylamine hydrochloride (0.575 g, 5.93 mmol), NMM (1.80 g, 17.8 mmol), HOBt (0.800 g, 5.93 mmol) and EDCI (1.25 g, 6.53 mmol). The reaction mixture was stirred for 1 h at 0°C. Water (20 mL) was added and the phases were separated. The organic phase was washed with 1M HCl (2 x 20 mL), water (20 mL), sat. NaHCO_3_ (2 x 20 mL) and brine (20 mL), dried over Na_2_SO_4_, filtered and concentrated under reduced pressure to provide 1.29 g (66%) of the title compound as a yellow solid. LC-MS (ESI, m/z): 330 [M+H]^+^. ^1^H NMR (300 MHz, DMSO-*d*_6_) δ 7.42 (s, 1H), 7.13 - 7.15 (d, *J* = 8.4 Hz, 1H), 4.44 - 4.50 (m, 1H), 3.72 (s, 3H), 3.05-3.20 (m, 5H), 1.99 - 2.24 (m, 2H), 1.85 - 1.97 (m, 1H), 1.69 - 1.83 (m, 1H), 1.58 - 1.69 (m, 1H), 1.37 - 1.44 (m, 11H).

***tert*-Butyl ((*S*)-4-(benzyloxy)-3-oxo-1-((*S*)-2-oxopiperidin-3-yl)butan-2-yl)carbamate (8)**

To a mixture of Mg (1.39 g, 57.3 mmol) and HgCl_2_ (1.04 g, 3.83 mmol) in THF (50 mL) cooled at -45°C was added benzylchloromethyl ether (8.99 g, 57.4 mmol). The mixture was stirred at -45-5°C for 5 h. After cooling to - 45°C, **7** (2.10 g, 6.37 mmol) was added. The reaction mixture was slowly allowed to warm to rt and was stirred at rt overnight. The reaction was quenched by addition of sat. NH_4_Cl (30 mL). The reaction mixture was extracted with EtOAc (3 x 50 mL). The organic phases were combined, washed with brine (2 x 50 mL), dried over Na_2_SO_4_, filtered and concentrated under reduced pressure. The residue was purified by flash chromatography on silica gel using a gradient of MeOH (0-5%) in CH_2_Cl_2_ to afford 700 mg (28%) of the title compound as a yellow oil. LCMS (ESI, m/z): 391 [M+H]^+^. ^1^H NMR (400 MHz, Chloroform-*d*) δ 7.45 - 7.29 (m, 5H), 6.03 (s, 1H), 5.90 - 5.92 (m, 1H), 4.47 - 4.72 (m, 3H), 4.21 - 4.42 (m, 2H), 3.27 - 3.30 (m, 2H), 2.30 - 2.42 (m, 1H), 2.04 - 2.21 (m, 2H), 1.80 - 1.92 (m, 2H), 1.65 - 1.77 (m, 1H), 1.48 - 1.57 (m, 1H), 1.44 (s, 9H).

**(*S*)-3-((*S*)-2-Amino-4-(benzyloxy)-3-oxobutyl)piperidin-2-one hydrochloride (9)**

To a solution of **8** (300 mg, 0.768 mmol) in dioxane (3 mL) cooled at 0°C was added 4N HCl in dioxane chloride (3.0 mL, 12.0 mmol). The reaction mixture was stirred at rt for 1 h and was concentrated under reduced pressure to afford quantitatively the title compound as a yellow solid. LC-MS (ESI, m/z): 291 [M+H]^+^.

**Scheme S3. Synthesis of ALG-097161*^a^***

*^a^*Reagents and conditions: (i) TCFH, NMI, CH_3_CN, 18%. (ii) H_2_, Pd/C, EtOH, 14%.

**(1*S*,3a*R*,6a*S*)-*N*-((*S*)-4-(Benzyloxy)-3-oxo-1-((*S*)-2-oxopiperidin-3-yl)butan-2-yl)-2-(4-methoxy-1*H*-indole-2-carbonyl)octahydrocyclopenta[*c*]pyrrole-1-carboxamide (10)**

To a mixture of **4** (250 mg, 0.861 mmol), **9** (250 mg, 0.861 mmol) and chloro-*N*,*N*,*N*′,*N*′-tetramethylformamidinium hexafluorophosphate (265 mg, 0.947 mmol) in acetonitrile (6 mL) cooled at 0°C was added NMI (353 mg, 4.31 mmol). The reaction mixture was stirred at rt for 1 h. The reaction mixture was diluted with water (3 mL) and extracted with EtOAc (3 x 20 mL). The organic phases were combined, washed with brine (2 x 20 mL), dried over Na_2_SO_4_, filtered and concentrated under reduced pressure. The residue was purified by preparative TLC using 5% MeOH in CH_2_Cl_2_ as eluent to afford 95 mg (18%) of the title compound as a yellow oil. LCMS (ESI, m/z): 601 [M+H]^+^.

**(1*S*,3a*R*,6a*S*)-*N*-((*S*)-4-Hydroxy-3-oxo-1-((*S*)-2-oxopiperidin-3-yl)butan-2-yl)-2-(4-methoxy-1*H*-indole-2-carbonyl)octahydrocyclopenta[*c*]pyrrole-1-carboxamide (ALG-097161)**

To a solution of **10** (90 mg, 0.150 mmol) in EtOH (5 mL) was added Pd/C (90 mg). The reaction mixture was stirred at rt overnight under H_2_ atmosphere. The reaction mixture was filtered through a celite pad and the solids were washed with EtOH (20 mL). The filtrate was concentrated under reduced pressure. The residue was purified by preparative HPLC to afford 11 mg (14%) of the title compound as a white solid. LCMS (ESI, m/z): 511 [M+H]^+^. ^1^H NMR (400 MHz, DMSO-*d_6_*, 80 °C) δ 11.21 (s, 1H), 8.38 (s, 1H), 7.04 - 7.13 (m, 3H), 6.87 (s, 1H), 6.49 - 6.51 (d, *J* = 7.2 Hz, 1H), 4.46 - 4.69 (m, 3H), 4.17 - 4.20 (m, 2H), 3.95-4.15 (m, 1H), 3.87 (s, 3H), 3.69 - 3.73 (m, 1H), 2.90-3.20 (m, 2H), 2.65 - 2.70 (m, 1H), 2.70 - 2.80 (m, 1H), 2.25 - 2.26 (m, 1H), 2.09 - 2.12 (m, 1H), 1.48 - 1.95 (m, 11H).
